# Male rat hypothalamic extraretinal photoreceptor Opsin3 is sensitive to osmotic stimuli and light

**DOI:** 10.1111/jne.13363

**Published:** 2024-01-09

**Authors:** Soledad Bárez‐López, Paul Bishop, Daniel Searby, David Murphy, Michael P. Greenwood

**Affiliations:** ^1^ Translational Health Sciences, Bristol Medical School University of Bristol Bristol UK; ^2^ Present address: Instituto de Investigaciones Biomédicas Consejo Superior de Investigaciones Científicas (CSIC)—Universidad Autónoma de Madrid (UAM) Madrid Spain

**Keywords:** hypothalamus, light‐sensitive, opsin, paraventricular nucleus, supraoptic nucleus

## Abstract

The light‐sensitive protein Opsin 3 (Opn3) is present throughout the mammalian brain; however, the role of Opn3 in this organ remains unknown. Since *Opn3* encoded mRNA is modulated in the supraoptic and paraventricular nucleus of the hypothalamus in response to osmotic stimuli, we have explored by in situ hybridization the expression of *Opn3* in these nuclei. We have demonstrated that *Opn3* is present in the male rat magnocellular neurones expressing either the arginine vasopressin or oxytocin neuropeptides and that *Opn3* increases in both neuronal types in response to osmotic stimuli, suggesting that Opn3 functions in both cell types and that it might be involved in regulating water balance. Using rat hypothalamic organotypic cultures, we have demonstrated that the hypothalamus is sensitive to light and that the observed light sensitivity is mediated, at least in part, by Opn3. The data suggests that hypothalamic Opn3 can mediate a light‐sensitive role to regulate circadian homeostatic processes.

## 1 INTRODUCTION

Synchronisation to periodic cues such as food/water availability and light/dark cycles is crucial for homeostasis. Modern lifestyles disturb our diurnal rhythms, most acutely by jetlag, but more insidiously by shift work and exposure to artificial light at night (ALAN), with consequential increased susceptibility of night‐time workers to diabetes, obesity, cardiovascular disease and depression.^1^ It is, therefore, essential to explore the mechanisms of light‐regulated physiology and behaviour in order to improve health and well‐being. Traditionally, it was believed that opsins in the rods and cones and a subset of intrinsically photosensitive retinal ganglion cells were the only light‐sensitive proteins in mammals. Following the discovery of extraretinal light‐sensitive proteins in thornback ray and chicken pineal glands,^2^, ^3^ Encephalopsin or Opsin 3 (Opn3) was the first extraretinal opsin identified in the mammalian brain^4^ suggesting that light may play a broader functional role than previously recognised.

Opn3 is a seven‐transmembrane domain G‐protein‐coupled receptor that is activated by blue light^5^, ^6^ and can mediate light‐dependent^6^, ^7^ but also light‐independent processes.^8^ Opn3 has been found to activate Gi/Go‐type G proteins leading to decreases in cAMP.^5^, ^8^ In the embryonic mouse brain, Opn3 is observed from E9.5 in the ganglia that will develop into the trigeminal, facial and vestibulocochlear cranial nerves and, throughout brain development, its distribution increases in several motor‐sensory tracts.^9^ In the adult brain, *Opn3* RNA^4^ and Opn3 protein^10^, ^11^ are clearly detected in the thalamus, the cerebellum, portions of the frontal cortex, the hippocampus, the anterior medial preoptic areas and several hypothalamic nuclei including the paraventricular nucleus (PVN) and the supraoptic nucleus (SON). However, more than two decades after its discovery, Opn3 remains one of the least characterised opsins and its brain functions remain elusive.

In recent transcriptome studies, we observed that *Opn3* mRNA expression in the paraventricular (PVN) and the supraoptic nucleus (SON) are consistently modulated in response to osmotic hypo‐ and hypertonic challenges,^12^, ^13^, ^14^ suggesting that OPN3 might in some way be involved in regulating physiological or behavioural responses to perturbations in water balance. The PVN and SON are hypothalamic nuclei part of the hypothalamo‐neurohypophysial system (HNS), a neurosecretory apparatus responsible for the production of the peptide hormones arginine vasopressin (AVP) and oxytocin (OXT). The HNS consists of two distinct populations of magnocellular neurones (MCNs) in the SON and PVN that separately synthesise either AVP or OXT. MCN axons project to blood capillaries of the posterior pituitary gland neuro‐vascular interface.^15^ To counteract hyperosmotic imbalance, such as that evoked by chronic dehydration or salt‐loading,^12^ the HNS releases AVP and OXT into the bloodstream^16^ to regulate water balance. Moreover, MCNs also send extra‐neurohypophysial collateral projections to other brain areas that can potentially regulate behavioural responses to physiological challenges^17^ including social behaviour,^18^ stress response and energy balance.^19^

To gather insights regarding the potential role of Opn3 in the brain, in this work, we have evaluated if *Opn3* is expressed in both AVP and OXT neurones and whether it is responsive to osmotic challenges in both neuronal types. Furthermore, we have assessed whether the hypothalamus is sensitive to light and whether this sensitivity is mediated by Opn3.

## 2 MATERIALS AND METHODS

### 2.1 Animals

All experimental procedures involving animals were performed in strict accordance with the provision of the UK Animals (Scientific Procedures) Act (1986). The study was designed following the ARRIVE (Animal Research: Reporting of In Vivo Experiments) guidelines^20^ and carried out under Home Office UK licences (PPL 30/3278). All the protocols were approved by the University of Bristol Animal Welfare and Ethical Review Board.

Male Sprague–Dawley rats weighing 250–300 g (Envigo) were maintained under a 14:10 light–dark cycle (lights on 5.00 a.m.) at a constant temperature of 21–22°C and a relative humidity of 50%–60% (v/v). Animals were housed in groups of 2 or 3 with environmental enrichment consisting of nesting material, cardboard tube and a chew block with ad libitum access to food and water for 1 week prior to experimentation. The water deprivation (WD) protocol involved the removal of water for 72 h with ad libitum access remaining for controls. In the rehydration protocol, water was reintroduced following 72 h dehydration for 4, 8 and 24 h prior to sample collection. Rats were euthanised by cranium striking followed by guillotine decapitation and the brains were removed and immediately frozen in powdered dry ice. All samples were collected between 9.00 a.m. and 1.00 p.m.

### 2.2 RNAscope in situ hybridization

Fresh‐frozen brains were sliced into 16 μm coronal sections and directly mounted on Superfrost Plus slides (Thermo Fisher Scientific, Waltham, MA, USA) and stored −80°C. RNAscope in situ hybridization was performed as described^21^, ^22^ using the probes Rn‐AVP‐C2 (Advanced Cell Diagnostics, 401421‐C2) targeting 20–525 of rat *Avp* gene, Rn‐OPN3 (Advanced Cell Diagnostics, 578411) targeting 418–1702 of rat *Opn3* gene and Rn‐OXT‐C3 (Advanced Cell Diagnostics, 479631‐C3) targeting 3–493 of rat *Oxt* gene.

### 2.3 Adeno‐associated virus (AAV) production, purification and titration

A Opn3 short hairpin RNA (shRNA) (GGGACAGGCCAAAGAAGAAAG) and a non‐targeting shRNA sequence (AATTCTCCGAACGTGTCACGT),^23^ used as a control, were cloned into HuSH shRNA GFP AAV Cloning Vector (pGFP‐A‐shAAV Vector; TR30034, Origene). The efficiency of the specific *Opn3* shRNA was tested in HEK293T/17 cells (human embryonic kidney cell line, CRL‐11268, ATCC) transiently overexpressing rat *Opn3*. Virus particles were produced as previously described.^24^ Briefly, HEK293T cells were transiently transfected with the ITR‐flanked transgene together with three separate packaging plasmids including pHelper Vector (340202, Cell Biolabs), pAAV‐RC1 Vector (VPK‐421, Cell Biolabs) and pAAV‐RC2 Vector (VPK‐422, Cell Biolabs) using Lipofectamine 3000 Transfection Reagent (11668019, Invitrogen) according to the manufacture's specifications. 72 h after transfection, cells were harvested and lysed with 150 mM NaCl, 20 mM Tris pH 8.0, 0.5% (w/v) sodium deoxycholate and 50 units per ml of benzonase nuclease. Viral particles were purified using HiTrap heparin columns (GE HealthCare Life Science), concentrated with Amicon ultra‐4 centrifugal filters to 100 μL and diluted with an equal volume of sterile phosphate‐buffered saline (PBS). Titers of AAV‐shOpn3‐eGFP and AAV‐shNT‐eGFP vector stocks were determined by direct qPCR amplification.^25^

### 2.4 Hypothalamic organotypic cultures

Organotypic cultures were performed as previously described.^26^, ^27^ Sprague–Dawley pups at postnatal day 5–7 (Envigo) were decapitated, and the brains were extracted and incubated in cold‐Hank's solution for 5 min. A block containing the hypothalamus was dissected from the brain and placed onto a Mcllwain Tissue Chopper to obtain 400 μm sections containing the PVN. Sections were incubated in ice‐cold Hank's solution for 1 h and then placed onto a Millicell Cell Culture Insert (30 mm, hydrophilic PTFE, 0.4 μm; Millipore PICM03050) in a 6‐well tissue culture plate with 1.1 mL of standard culture medium containing Eagles basal medium 50% (v/v), Heat inactivated horse serum 25% (v/v) (Gibco, 26050070), Hanks balanced salt solution 25% (v/v), 1 mM glutamine (Gibco, 35050038), 5 mg/mL glucose (Sigma, G5400), 25 μg/mL penicillin and 25 μg/mL streptomycin. The following day, sections containing the PVN were identified under a bright field microscope and for hypothalamic cultures treated with AAVs, 1 μL containing either 1.59 × 10^9^ viral particles of AAV‐shOpn3‐eGFP or 1.31 × 10^9^ viral particles of AAV‐shNT‐eGFP was placed on top of the PVN, which spread throughout the section. The culture sections were incubated at 37°C, 5% (v/v) CO_2_ for 14 days before performing an experiment, replacing the culture medium every 2 days. 4 days prior to the experiment, the standard medium was replaced with serum‐free medium.

On the day of the experiment, the hypothalamic cultures were placed inside black boxes with and without light‐emitting diodes (LEDs) capable of emitting 450–550 nm light at a 144 ± 11 lux intensity, as described,^7^ for 2 h at 37°C, 5% (v/v) CO_2_. Temperature recordings obtained by placing a thermometer inside the black boxes revealed no variations in temperature attributable to light exposure. Following darkness/light exposure, the sections were washed three times in PBS, fixed in 4% paraformaldehyde for 20 min and washed three times in PBS prior to immunolabelling.

### 2.5 Immunolabelling

The tissues fixed on Millicell Cell Culture Inserts were blocked in PBS containing 0.1% Triton X‐100, 4% bovine serum albumin (BSA) and 5% donkey serum at RT for 1 h and incubated overnight at 4°C with a primary antibody against cFOS (1:100; Santa Cruz Biotechnology, sc‐166940) in PBS containing 0.1% (v/v) Triton X‐100, 4% (w/v) BSA and 1% (v/v) donkey serum. The following day, the tissues washed in PBS and incubated with the secondary antibody donkey anti‐mouse Alexa Fluor Plus 647 (1:500, Invitrogen, A‐31573) in PBS containing 0.1% (v/v) Triton, 4% (w/v) BSA and 1% (v/v) donkey serum at RT for 1 h. The tissues were then washed in PBS and incubated with 4′,6‐diamidino‐2‐phenylindole dihydrochloride (DAPI, D1306; Molecular Probes) in PBS. Finally, the tissues with the Millicell Cell Culture Inserts were mounted with ProLong Gold Antifade Mountant (Invitrogen, P36930).

### 2.6 Image acquisition and data analysis

Images from RNAscope in situ hybridization studies were acquired with a Leica SP5‐II AOBS confocal laser scanning microscope attached to a Leica DMI 6000 inverted epifluorescence microscope using a 63× PL APO CS lens with a 3.4‐zoom factor. Quantification of Opn3 RNA dots in the nucleus (DAPI labelling close to either AVP‐ or OXT‐positive cytoplasm) or cytoplasm (AVP or OXT labelling) of AVP or OXT neurones was performed in a single 63× plane with a 3.4‐zoom factor using a modular workflow plugin for Fiji created by Dr Stephen J Cross from the Wolfson Bioimaging Facility of the University of Bristol, as described.^21^ Images from immunofluorescence studies were acquired with a Leica SP8 AOBS confocal laser scanning microscope attached to a Leica DM I8 inverted epifluorescence microscope using a 20× HC PL APO CS2 lens. Percentage of cFOS positive (cFOS+) cells were calculated by quantifying the number of cFOS+ nuclei in relation to the number of total DAPI‐stained nuclei on the entire image acquired with 20× PL APO CS lens using Fiji. Raw image files were processed to generate composite images using Fiji.

### 2.7 Statistical analysis

Data are presented in box and whiskers plots. Statistical analyses were performed with GraphPad Prism software v8. Normality of the data was assessed by the Shapiro–Wilk test. Means between two groups were compared by two‐tailed unpaired Student's *t*‐test and differences between means for more than two groups was performed by one‐way analysis of variance (ANOVA) and the Tukey's post hoc test to correct for multiple comparisons. Statistical significance was set at *p* < .05.

## 3 RESULTS

### 3.1 Opn3 mRNA is expressed in AVP and OXT neurons and increases in response to osmotic stimulation

In order to get further information regarding the cell types expressing *Opn3* in the HNS and its responses to stimuli, we performed multiplex RNAscope in situ hybridization labelling of *Opn3* transcripts in combination with the *Avp* and *Oxt* mRNAs to identify AVP and OXT MCNs. We explored *Opn3* expression in male euhydrated control rats, male rats following 72 h of WD and 4, 8 and 24 h of rehydration after WD. To assess potential *Opn3* mRNA nuclear retention in response to osmotic stimulation, as we have previously observed for another osmotic‐responsive gene *Caprin2*,^21^ *Opn3* mRNA expression was differentially assessed in the nucleus and the cytoplasm.

**FIGURE 1:**
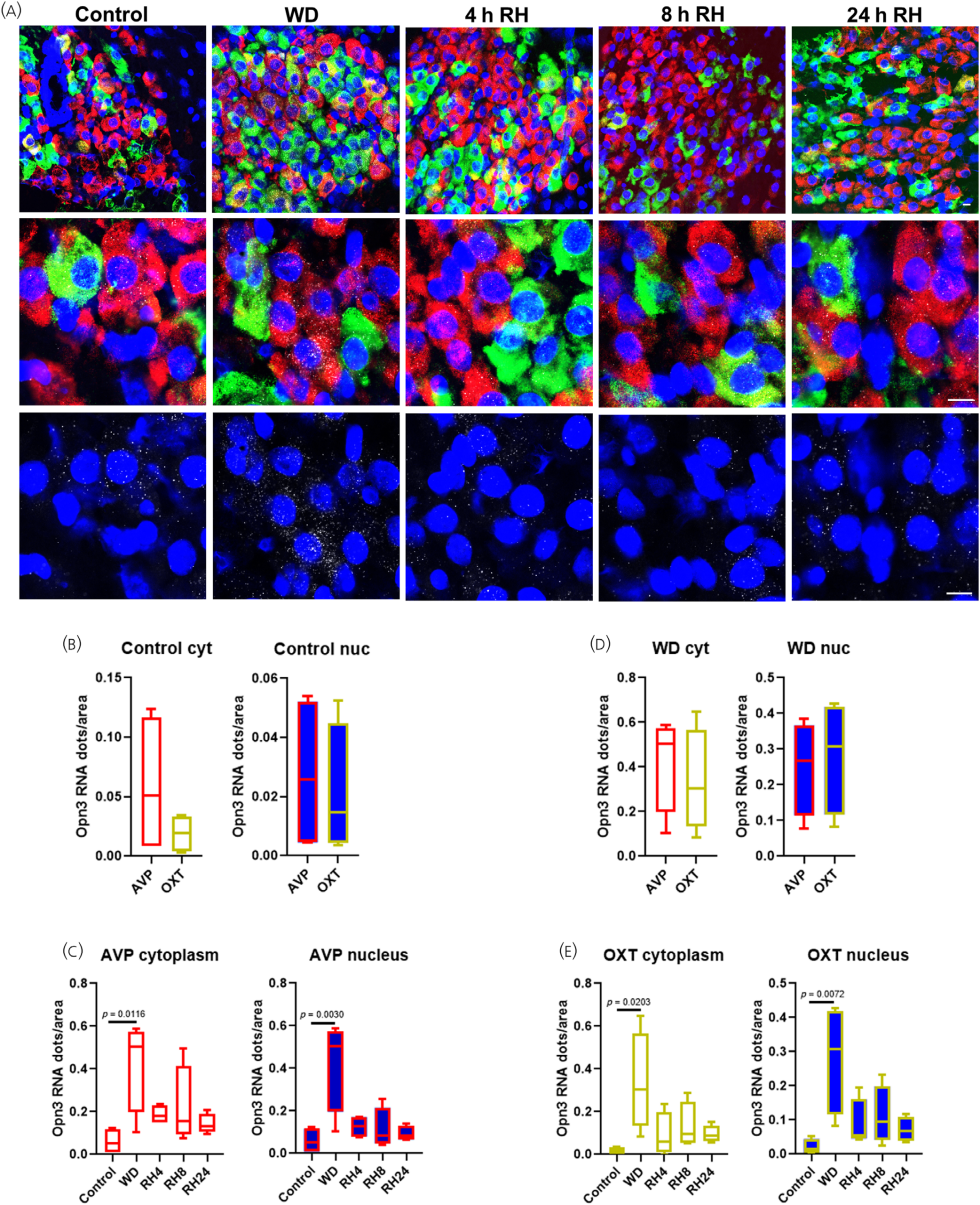
*Opn3* mRNA expression in the supraoptic nucleus (SON). DAPI (blue), *Avp* (red), *Oxt* (green) and *Opn3* (grey) labelled by RNAscope in situ hybridisation in control (*n* = 4 rats) conditions, after 72 h of water deprivation (WD; *n* = 4 rats), after 72 h of WD followed by 4 h rehydration (4 h RH; *n* = 4 rats), 8 h rehydration (8 h RH; *n* = 4 rats) or 24 h rehydration (24 h RH; *n* = 4 rats). (A). Graphs showing gene expression as a function of *Opn3* mRNA dots/cytoplasm of AVP vs. OXT neurones in control conditions (B), *Opn3* mRNA dots/nuclei of AVP vs. OXT neurones in control conditions (C), *Opn3* mRNA dots/cytoplasm of AVP vs. OXT neurones in WD conditions (D), *Opn3* mRNA dots/nuclei of AVP vs. OXT neurones in WD conditions (E), *Opn3* mRNA dots/cytoplasm AVP neurones (F), *Opn3* mRNA dots/nuclei AVP neurones (G), *Opn3* mRNA dots/cytoplasm OXT neurones (H), *Opn3* mRNA dots/nuclei OXT neurones (I). Data are presented in box‐plots representing the 25th (bottom), 50th (middle‐line) and 75th (top) quartiles with whiskers extending from minimum to maximum values. Differences between means in B and C were determined by two‐tailed unpaired Student's *t*‐test and in C and E by one‐way analysis of variance (ANOVA) and the Tukey's post hoc test to correct for multiple comparisons. Scale bar represents 10 μm.

In the SON, analysis of *Opn3* transcript expression in euhydrated conditions revealed that *Opn3* is found in both cytoplasmatic and nuclear compartments of AVP and OXT MCNs to the same extent (Figure 1A, B; t(6) = 1.294, *p* = .2433, unpaired *t*‐test in cytoplasm and t(6) = .3607, *p* = .7306, unpaired test in nucleus). WD increased *Opn3* abundance in the cytoplasm and nucleus of both AVP and OXT neurones (Figure 1A, C, E; AVP cytoplasm F(4, 15) = 4.113, *p* = .0191, one‐way ANOVA, Tukey post‐hoc test control vs. WD *p* = .0116; AVP nucleus F(4, 15) = 6.815, *p* = .0025, one‐way ANOVA, Tukey post‐hoc test control vs. WD *p* = .0030; OXT cytoplasm F(4, 15) = 3.633, *p* = .0292, Tukey post‐hoc test control vs. WD *p* = .0203; OXT nucleus F(4, 15) = 4.850, *p* = .0104, Tukey post‐hoc test control vs. WD *p* = .0072). There were no differences in the expression of *Opn3* between AVP and OXT neurons following WD (Figure 1A, D; t(6) = .5622, *p* = .5944, unpaired t‐test in cytoplasm and t(6) = .3607, *p* = .7306, unpaired t‐test in nucleus). During rehydration, *Opn3* expression decreased towards control levels in the cytoplasm and nucleus of AVP and OXT cells as early as 4 h following water reintroduction (Figure 1A, C, E).

**FIGURE 2:**
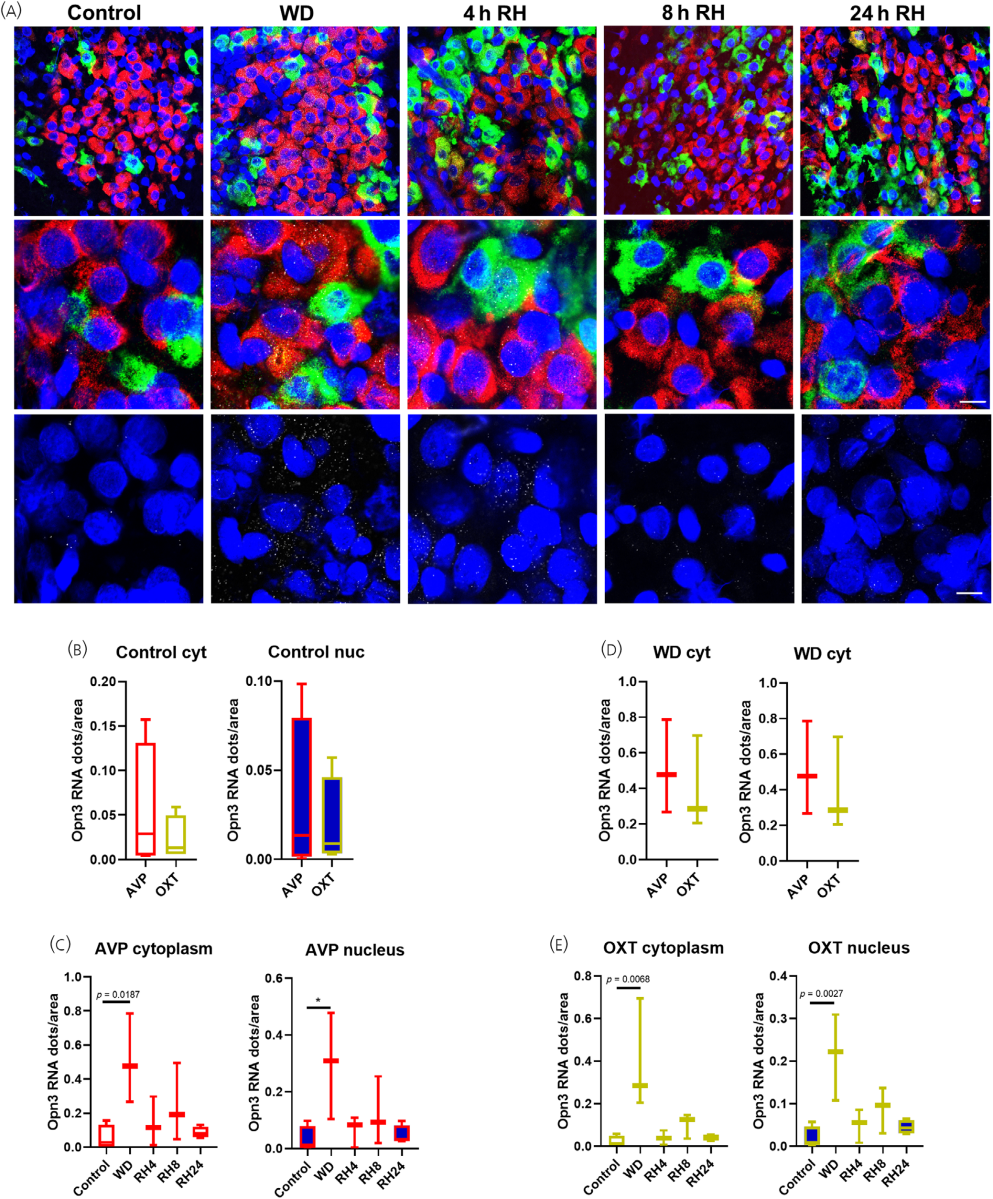
*Opn3* mRNA expression in the paraventricular nucleus (PVN). DAPI (blue), *Avp* (red), *Oxt* (green) and *Opn3* (grey) labelled by RNAscope in situ hybridisation in control (*n* = 4 rats) conditions, after 72 h of water deprivation (WD; *n* = 4 rats), after 72 h of WD followed by 4 h rehydration (4 h RH; *n* = 4 rats), 8 h rehydration (8 h RH; *n* = 4 rats) or 24 h rehydration (24 h RH; *n* = 4 rats). Magnocellular neurones were distinguished from parvocellular based on their specific locations. (A). Graphs showing gene expression as a function of *Opn3* mRNA dots/cytoplasm of AVP vs. OXT neurones in control conditions (B), *Opn3* mRNA dots/nuclei of AVP vs. OXT neurones in control conditions (C), *Opn3* mRNA dots/cytoplasm of AVP vs. OXT neurones in WD conditions (D), *Opn3* mRNA dots/nuclei of AVP vs. OXT neurones in WD conditions (E), *Opn3* mRNA dots/cytoplasm AVP neurones (F), *Opn3* mRNA dots/nuclei AVP neurones (G), *Opn3* mRNA dots/cytoplasm OXT neurones (H), *Opn3* mRNA dots/nuclei OXT neurones (I). Data are presented in box‐plots representing the 25th (bottom), 50th (middle‐line) and 75th (top) quartiles with whiskers extending from minimum to maximum values. Differences between means in B and C were determined by two‐tailed unpaired Student's *t*‐test and in C and E by one‐way analysis of variance (ANOVA) and Tukey's post hoc test to correct for multiple comparisons. Scale bar represents 10 μm.

Similar results were found in the PVN where *Opn3* expression was identified in both cytoplasmatic and nuclear compartments of AVP and OXT MCNs to the same extent in euhydrated control conditions (Figure 2A, B; t(6) = .8408, *p* = .4327, unpaired *t*‐test in cytoplasm and t(6) = .4628, *p* = .6598, unpaired test in nucleus). WD increased *Opn3* abundance in the cytoplasm and nucleus of both AVP and OXT neurones (Figure 2A, C, E; AVP cytoplasm F(4, 12) = 4.372, *p* = .0207, one‐way ANOVA, Tukey post‐hoc test control vs. WD *p* = .0187; AVP nucleus F(4, 12) = 3.960, *p* = .0283, one‐way ANOVA, Tukey post‐hoc test control vs. WD *p* = .0265; OXT cytoplasm F(4, 12) = 6.140, *p* = .0063, Tukey post‐hoc test control vs. WD *p* = .0068; OXT nucleus F(4, 12) = 7.065, *p* = .0037, Tukey post‐hoc test control vs. WD *p* = .0027), with no differences between AVP and OXT neurons following WD (Figure 2A, D; t(4) = .5369, *p* = .6198, unpaired *t*‐test in cytoplasm and t(4) = .6858, *p* = .5305, unpaired *t*‐test in nucleus). Rehydration decreased *Opn3* expression values to control levels in the cytoplasm and nucleus of AVP and OXT cells following 4 h of water reintroduction (Figure 2A, C, E).

### 3.2 Hypothalamic neurones are sensitive to blue light

**FIGURE 3:**
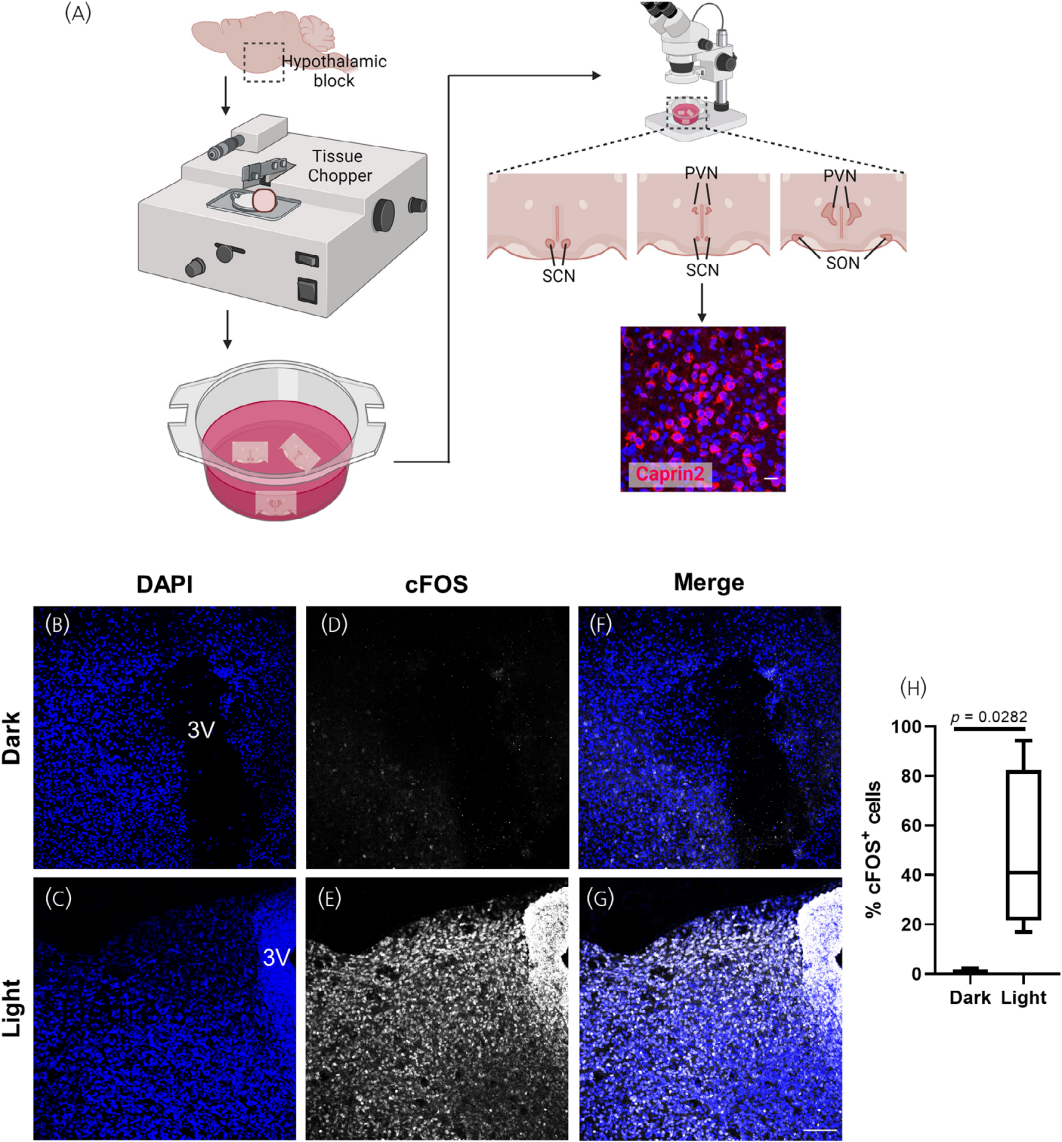
Hypothalamic organotypic cultures under darkness or blue light exposure. Workflow illustrating the generation of organotypic cultures. Brain samples from rat Sprague–Dawley pups at postnatal day 5–7 were dissected to obtain a hypothalamus‐containing block. This block was placed onto a Mcllwain Tissue Chopper to obtain 400 μm sections containing the PVN that were subsequently placed onto a Millicell Cell Culture Insert with standard culture medium. The following day, the sections were examined under bright field microscope to identify those sections containing the PVN. Some sections were immunolabelled against the magnocellular neuronal marker Caprin2, to verify the survival of magnocellular neurones (A). DAPI‐stained nuclei of PVN‐containing organotypic cultures incubated for 2 h in darkness (B) or under blue light exposure (C). cFOS immunolabelled cells in PVN‐containing organotypic cultures incubated for 2 h in darkness (D) or under blue light exposure (E). Merge of DAPI and cFOS immulabelled cells in PVN‐containing organotypic cultures incubated for 2 h in darkness (F) or under blue light exposure (G). (H) Percentage of cFOS positive cells (cFOS+) in PVN‐containing organotypic cultures incubated for 2 h in darkness (*n* = 4) or under blue light exposure (*n* = 4). One PVN‐containing section from each animal was used for the analysis. Data are presented in box‐plots representing the 25th (bottom), 50th (middle‐line) and 75th (top) quartiles with whiskers extending from minimum to maximum values. Differences between means in G were determined by two‐tailed unpaired Student's *t*‐test. 3 V, third ventricle. Scale bar represents 100 μm.

To investigate whether the hypothalamus is sensitive to blue light, we cultured organotypic cultures derived from rat hypothalamus inside black boxes with and without blue LEDs. Hypothalamic organotypic cultures containing the PVN were cultured in standard medium for 10 days and for 4 further days in serum‐free medium (Figure 3A). On the day of the experiment, cultures were incubated in the boxes with or without LED light exposure for 2 h, following which the cultures were fixed and immunostained for the activity‐induced immediate early gene cFOS. Hypothalamic cultures incubated in dark boxes presented 0.7549% ± 1.063 cFOS+ cells (Figure 3D, F) whereas hypothalamic cultures exposed to blue light significantly increased the number of cFOS‐expressing cells to 48.26% ± 33.02 (Figure 3E, G, H; T(6) = 2.876, *p* = .0282), indicating that hypothalamic cultures containing the PVN are sensitive to blue light.

**FIGURE 4:**
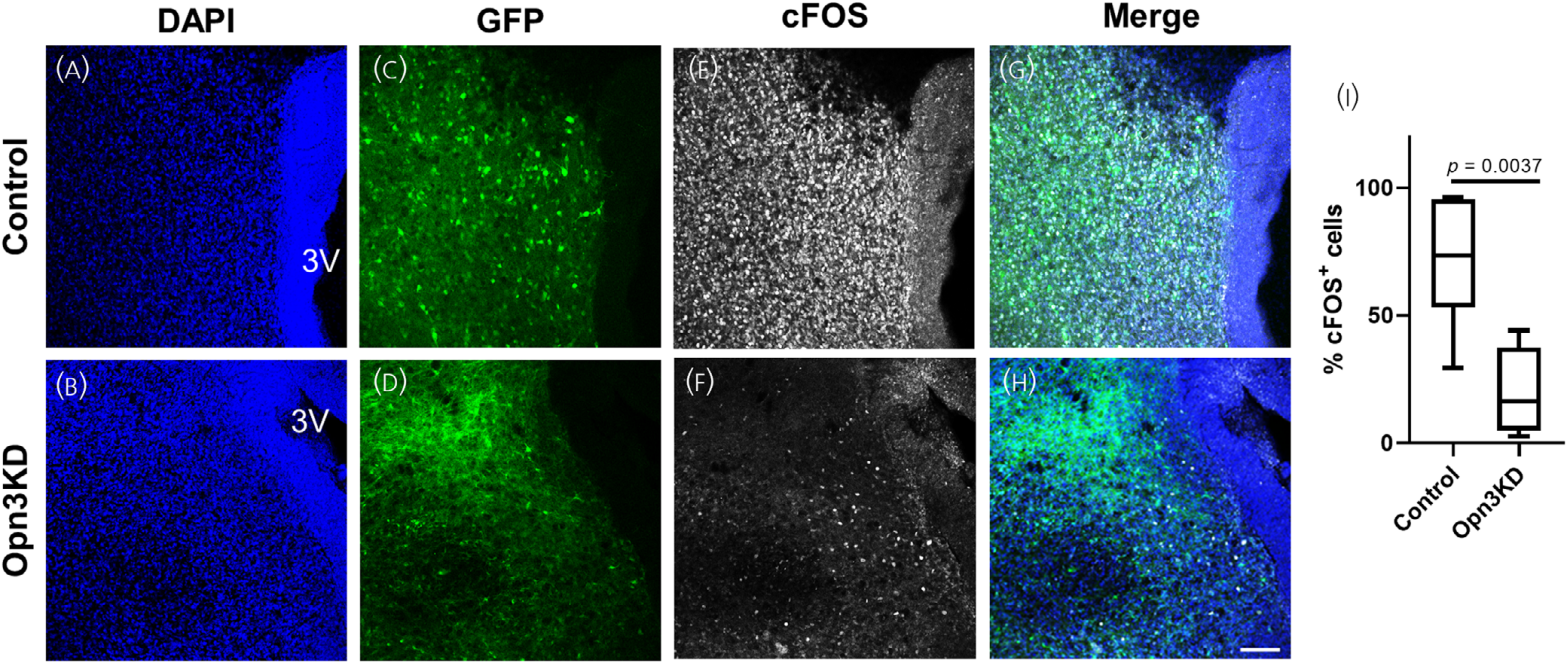
Control and Opn3 knockdown hypothalamic organotypic cultures under blue light exposure. Control (treated with a non‐target scrambled shRNA an eGFP tag) and Opn3 knockdown (Opn3KD, treated with specific Opn3 shRNA and an eGFP tag) PVN‐containing organotypic cultures incubated for 2 h under blue light exposure showing DAPI‐stained nuclei (A control, B Opn3KD); GFP expression (C control, D Opn3KD), cFOS immunolabelled cells (E control, F Opn3KD); and merge (G control, H Opn3KD). (I) Percentage of cFOS positive cells (cFOS+) in control (*n* = 6) and Opn3KD (*n* = 5) PVN‐containing organotypic cultures incubated for 2 h under blue light exposure. One PVN‐containing section from each animal was used for the analysis. Data are presented in box‐plots representing the 25th (bottom), 50th (middle‐line) and 75th (top) quartiles with whiskers extending from minimum to maximum values. Differences between means in I were determined by two‐tailed unpaired Student's *t*‐test. 3V, third ventricle. Scale bar represents 100 μm.

To assess whether the hypothalamic sensitivity to blue light is mediated by Opn3, we generated AAV vectors containing a specific Opn3 shRNA and an eGFP tag (AAV‐shOpn3‐eGFP) and AAV vectors containing a non‐target scrambled shRNA and an eGFP tag (AAV‐shNT‐eGFP) as controls. In this experiment, we derived organotypic cultures from rat hypothalamus and the following day placed the AAV‐shOpn3‐eGFP vector (Opn3 knock down, Opn3KD) or the AAV‐shNT‐eGFP vector (control) on top of the PVN. Following 10 days of incubation, we replaced the standard medium with serum‐free medium for four further days. On the day of the experiment, we placed both control and Opn3KD hypothalamic cultures in black boxes containing blue LEDs. After 2 h of light exposure, the cultures were fixed and immunostained for cFOS. We observed that, both control and Opn3KD cultures expressed GFP indicating successful viral transduction (Figure 4C, D). In addition, whilst control hypothalamic cultures retained the ability to respond to blue light, as observed by 71.45% ± 24.81 cFOS positive cells (Figure 4E, G), only 20.27 ± 17.19 cells in Opn3KD cultures were positive for cFOS (Figure 4F, H), indicating a blunted response to light in Opn3KD hypothalamic cultures in comparison to controls (Figure 4I, T(9) = 3.886, *p* = .0037).

## 4 DISCUSSION

As a first step to unravelling the role of Opn3 in the HNS, in this work we have identified AVP and OXT neurones as hypothalamic cell types expressing *Opn3*. We found that *Opn3* is present to the same extent in both AVP and OXT neurones and that *Opn3* expression increases in both neuronal types to the same degree in response to osmotic stimulation, suggesting a role for Opn3 in both neuronal types in the SON and the PVN. Since the peptide hormones AVP and OXT exert well‐known roles in the control of water reabsorption and natriuresis, respectively, at the level of the kidney,^28^ it is tempting to speculate that Opn3 might be involved in regulating water balance. In relation to this, Opn3‐deficient mice display decreased food and water intake,^29^ which could potentially be related to the role of Opn3 in osmotic homeostasis. It has long been known that AVP and OXT show daily rhythms of secretion^30^, ^31^, ^32^, ^33^, ^34^ that can be affected by constant light.^35^ The fact that Opn3 is a light‐sensitive protein raises interesting possibilities, such as the presence of intrinsic circadian control mechanisms directly in the HNS.

One essential question is whether OPN3 mediates a light‐sensitive role in the brain, and in particular, the hypothalamus. Our findings indicate that neurones from the PVN are sensitive to light, as seen by the increases in the expression of the activity‐induced immediate early gene cFOS when exposing hypothalamic organotypic cultures to blue light. Moreover, this hypothalamic light‐sensitivity seems to be mediated, at least in part, by Opn3, as knocking down Opn3 in hypothalamic organotypic cultures decreases the responses in cFOS activation when exposed to blue light. It is worth taking into consideration that, even though the present analysis focused on the PVN, it is not possible to fully rule out that neuronal connections from other hypothalamic areas remaining in the organotypic slides (i.e., the SNC) that project to the PVN could be partly mediating the Opn3‐mediated light‐sensitive responses observed in the PVN. Nevertheless, while it is important to take the inherent complexity of neuronal connectivity within the hypothalamus into consideration, the present findings strongly indicate a light‐sensitive role of Opn3 in the hypothalamus. The next question is whether hypothalamic Opn3 can regulate light‐sensitive physiological processes. In relation to this, there is increasing evidence supporting that light can penetrate the skull and alter neuronal activity. For instance, it has been demonstrated that external extraocular light can activate brain photoreceptors.^36^ In addition, transcranial light has also been shown to modulate Opn3 expression and neurotransmitter release in the hypothalamus.^37^ All these data suggest that hypothalamic‐dependent homeostatic processes could be indeed directly modulated by light.

In addition to regulating water balance, Opn3 in the HNS could be regulating other physiological processes. For instance, Opn3‐deficient mice have diminished thermogenesis during cold exposure.^29^ Since, endogenous AVP in the brain has been suggested to mediate antipyretic effects^38^ and central infusion of AVP has been shown to produce hypothermia,^39^ Opn3 could be involved in body temperature regulation through AVP production. Interestingly, the delivery of blue light onto the mouse SON has been shown to promote a significant arousal effect,^40^ suggesting that hypothalamic Opn3 might be involved in regulating sleep and arousal.

In summary, we have produced evidence that Opn3 in the HNS is sensitive to osmotic stimuli and light. More than 20 years after the discovery of Opn3 in the brain, its role in this organ remains unknown. We propose that hypothalamic Opn3 can play a light‐sensitive role in the regulation of circadian homeostatic processes such as for example water balance, body temperature and sleep. Further studies should be performed to fully elucidate the role of Opn3 in the brain and its potential light‐mediated effects. Of particular interest would be to assess the specific role of Opn3 in mediating the light‐sensitive responses particularly in AVP or OXT neurones by knocking down Opn3 specifically in these cell types using specific AVP and OXT promoter.^41^ Furthermore, given that Opn3 responses to osmotic stimuli have exclusively been evaluated in male rats and acknowledging the established sex differences in OXT and AVP neurones,^42^, ^43^ it is important to investigate whether these findings can be extrapolated to females. Such investigations are essential to gain a better understanding of the impact of circadian rhythm disruption by light on our health and well‐being.

## AUTHOR CONTRIBUTIONS

**Soledad Bárez‐López:** Conceptualization; data curation; formal analysis; funding acquisition; investigation; methodology; project administration; resources; validation; visualization; writing – original draft. **Paul Bishop:** Formal analysis; investigation; methodology; validation; writing – review and editing. **Daniel Searby:** Data curation; formal analysis; investigation; methodology; writing – review and editing. **David Murphy:** Conceptualization; data curation; formal analysis; funding acquisition; investigation; methodology; project administration; resources; supervision; validation; visualization; writing – review and editing. **Michael P. Greenwood:** Conceptualization; data curation; formal analysis; funding acquisition; investigation; methodology; resources; supervision; validation; visualization; writing – review and editing.

## CONFLICT OF INTEREST STATEMENT

The authors declare that they have no conflicts of interest.

### PEER REVIEW

The peer review history for this article is available at https://www.webofscience.com/api/gateway/wos/peer-review/10.1111/jne.13363.

## Data Availability

All data are available in the manuscript. Any additional information is available from the lead contact upon request.
